# Defective Teeth and Nutrition

**Published:** 1923-03

**Authors:** 


### DEFECTIVE TEETH AND NUTRITION

The development and maintenance of good teeth are not the professional concern of the dentist alone, they are of equal moment to the physician. For the lack of more adequate or sympathetic consideration of dental problems on our part, the current points of view of our dental colleagues may be, in some measure, responsible. They have mainly, of late, regarded dental decay as a purely local affair, and have placed emphasis on the environment of the teeth far more than on their structure and nutrition. Hence the widespread emphasis on oral hygiene. In essence, it is taught that films forming on the tooth surfaces inclose bacteria and particles of carbohydrate food, which undergo fermentation, resulting in the formation of lactic acid. The latter, it is alleged, may then dissolve the lime salts on the surface of the teeth, leaving the organic matter to be attacked by putrefactive bacteria. Presently a cavity is formed which becomes a pocket full of menacing contents. The bacteriologic interpretation is thus placed foremost; and the consequent propaganda alleges that "a clean tooth never decays."

There are indications of a reaction against this preeminently environmental consideration of the subject of defective teeth. It was conspicuous in the meetings of the Section on Stomatology at the St. Louis session of the American Medical Association. The failure of experiment to support the hypothesis of damage from fermenting carbohydrates was pointed out by Howe. Animals fed on a diet to which were added large amounts of sugars—glucose, fructose, lactose and sucrose—dextrin and white flour, showed no dental effects at the end of a year. The sugars and starches adhered to the teeth constantly, and bacterial examination disclosed a fermentative flora; but no dental defects could be detected. The vigorous disputes as to the relative scientific merits of mouth washes and dentifrices of widely varying reaction and composition also attest the great uncertainty that still prevails.

As there is an unquestionable resemblance between the teeth and the bones in structure and chemical composition, and since the processes of calcification are analogous in them, it seems logical to apply the growing knowledge of bone pathology to the care of the teeth. The recent intensive study of rickets, especially in this country, by the method of dietary experiments on animals, may point the way to progress in dental pathology. No consideration of the physiology of bone can leave out of consideration the role of calcium and phosphorus in nutrition; but, in addition to these there is reason to assume that other factors comparable to the vitamins are operative in determining the proper deposition of the inorganic components in their organic matrix. Even with somewhat unfavorable proportions of calcium and phosphorus in the diet, the undetermined ''something'' in cod liver oil facilitates proper bone growth and function, as happens when it averts or cures rickets. Furthermore, as Shipley pointed out, in harmony with numerous reports in *The Journal,* animals and human beings as well, are enabled to compensate for faulty calcium-phosphorus ratios in the diet if they are exposed to the rays of the sun or the iron-chromium or cadmium arcs, or to the light of the mercury vapor quartz lamp. The fact that the beneficial rays from these sources may be screened off by ordinary window glass would seem to indicate that the active rays are in the ultra-violet spectrum. Again, since healing is more rapid under the influence of the cadmium arc than under a chromium iron or copper arc of equal intensity, the shorter ultra-violet rays (about 22,100 Angstrom units), probably have the greatest effect.

Through intentional variations in their rations, the internal structure of the skeletons of animals can now be altered experimentally. Depending on the factors affected, the resultant pathologic picture may be variable. Sometimes the result is identical with human rickets; again, there may be osteoporosis or still again a so-called osteosclerosis. Pseudorachitic conditions due to an overproduction of osteoid material arise when diets are deficient in calcium alone. By the use of suitable diets, animals can be reared through generations without caries-like lesions, pulp exposure, or maxillary defects. On the other hand, according to the reports of Baltimore investigators, it is possible to produce oral defects in the same species by dietary alterations. The percentage of such defects is greatest, in the case of rats, in those fed diets deficient in protein, calcium and vitamin A. It is believed that a deficiency of the antiscorbutic substance from the diet of man would no doubt be a factor in the production of oral disease. McCollum and his co-workers also suspect that severe oral disease may result from diets which are only relatively defective, in which the disturbance appears to be out of all proportion to the cause. They are careful to admit that it is not possible at this time to name any one deficiency which specifically causes dental or oral disease. The promise that lies in the further study of dental defects from the standpoint of dietary factors is evident. The results are almost certain to afford important contributions to the subject. A danger lies in one-sided points of view.

It must be remembered that malfunction of the endocrine glands has been enthusiastically advocated as the etiologic factor in dental caries; these assertions are based on clinical observations of hypoplastic and dystrophic teeth in cretins and in patients with acromegaly. Hammett has reported apparently specific dental defects following parathyroidectomy and associates them with consequent disturbances of calcium metabolism. These are days of ignorance as well as knowledge, when extremes of statement should still be avoided; hence, despite our enthusiasm for the newer findings, we can not yet accept the statements of those who aver that the American diet of the present time is "perilously near the danger mark" or "dangerously approaches the level of dietary deficiency." Some day, perhaps soon, we shall have a better knowledge.—Given in the *Journal of the American Medical Association,* Dec. 9, 1922.

